# Correction: Two-carbon ring expansion of bicyclic aziridines to oxazocines *via* aryne insertion into a σ C–N bond

**DOI:** 10.1039/d5sc90235e

**Published:** 2025-10-27

**Authors:** Daniel S. Rampon, Tuan Anh Trinh, Yekun Pan, Sierra Thein, Jacob W. Kailing, Ilia A. Guzei, Israel Fernández, Jennifer M. Schomaker

**Affiliations:** a Department of Chemistry, University of Wisconsin Madison Wisconsin 53706 USA schomakerj@chem.wisc.edu; b Departamento de Química Orgánica I and Centro de Innovación en Química Avanzada (ORFEO-CINQA), Facultad de Ciencias Químicas, Universidad Complutense de Madrid Madrid 28040 Spain israel@quim.ucm.es

## Abstract

Correction for ‘Two-carbon ring expansion of bicyclic aziridines to oxazocines *via* aryne insertion into a σ C–N bond’ by Daniel S. Rampon *et al.*, *Chem. Sci.*, 2025, https://doi.org/10.1039/d5sc04998a.

The authors regret that incorrect details were given for ref. 27 in the original article. The corrected version of ref. 27 can be found below as ref. 1.

The Royal Society of Chemistry apologises for these errors and any consequent inconvenience to authors and readers.
